# Real-time monitoring of tumor progression and drug responses in a preclinical mouse model of prostate cancer

**DOI:** 10.18632/oncotarget.8846

**Published:** 2016-04-20

**Authors:** Peng Xu, Naijin Xu, Kai Guo, Abai Xu, Fumiaki Takenaka, Eiji Matsuura, Chunxiao Liu, Hiromi Kumon, Peng Huang

**Affiliations:** ^1^ Department of Urology, Zhujiang Hospital, Southern Medical University, Guangzhou, People's Republic of China; ^2^ Department of Urology, Okayama University Graduate School of Medicine, Dentistry and Pharmaceutical Sciences, Okayama, Japan; ^3^ Okayama Medical Innovation Center, Okayama University, Okayama, Japan; ^4^ Innovation Center Okayama for Nanobio-Targeted Therapy, Okayama University Graduate School of Medicine, Dentistry and Pharmaceutical Sciences, Okayama, Japan

**Keywords:** prostate cancer, bioluminescence imaging, prostate specific antigen, immunotherapy, preclinical model

## Abstract

Monitoring disease progression through imaging is playing an increasingly important role in the treatment of prostate cancer. Here, we report that primary mouse prostate cancer cell lines stably expressing luciferase and tumor biomarkers can be monitored through bioluminescence imaging along with assays of serum biomarkers and immune function. Tumorigenesis in immunocompetent C57BL/6 mice can be monitored in by collecting samples from the dorsal flank, dorsolateral prostate, and tail vein to obtain real-time subcutaneous, orthotopic, and metastasis indicators, respectively. We used this technique to confirm the therapeutic effect of immune checkpoint blockade. Our findings suggest the presented indicators are ideally suited for real-time tracking of drug responses, tumor progression and immune function.

## INTRODUCTION

Cancer is a major public health problem in the world. Prostate cancer is a major cause of death and the second leading cause of cancer-related death in men. In 2014, an estimated 233,000 new cases of prostate cancer, with 29,480 cancer-related deaths, were reported in the United States [1, 2]. Although surgery can effectively control prostate cancer in the early stages, many newly diagnosed patients already have distant metastasis. Despite surgical treatment, androgen deprivation, radiation therapy, and chemotherapy, disease progression and metastases still occur in most cases [3, 4]. Therefore, there is an urgent need to understand the mechanisms underlying metastatic prostate cancer and to develop new and effective therapies.

In recent years, the increasing interest in the field of tumor immunotherapy is being driven by several remarkable breakthroughs. In particular, immune checkpoint blockade initiated a new paradigm shift in immunotherapy for cancer [5, 6]. However, monitoring and intravital imaging of cancer and its immune function is challenging, particularly in the setting of immunotherapy for early neoplasia.

Animal models provide the essential link between *in vitro* experiments and the development of novel therapeutic strategies by clinical studies [7]. In these models, the relationship between the host and tumor, processes of tumor invasion and metastasis, and effectiveness of different treatment measures can be evaluated. Xenografts are the most widely used model to help predict antitumor efficacy for specific disease types in a preclinical setting [7, 8]. However, existing models have not been able to simulate the entire process of tumorigenesis in humans, including development and metastasis [9].

A number of preclinical models in immunocompetent animals are currently used to study cancer growth and metastasis. Moreover, bioluminescent imaging in orthotopic and lung metastasis models has been reported for other cancers. However, in these immunocompetent animal models, never tumor biomarkers could be detected by laboratory examination. Therefore, there is a need for cancer models that combine imaging, tumor biomarker, and immune function measurement to allow instant tumor tracking and evaluation of new drug therapies. This model must be reliable, sensitive, and should take into account tumor progression, bioluminescence, and immune function [8–10]. In comparison with traditional methodologies, this biomarker/imaging-based approach could lead to improved, early, and sensitive assessment of the tumor status.

## RESULTS

### Generation and characterization of RM9-Luc-pIRES-KLK3 cells

**Figure 1:**
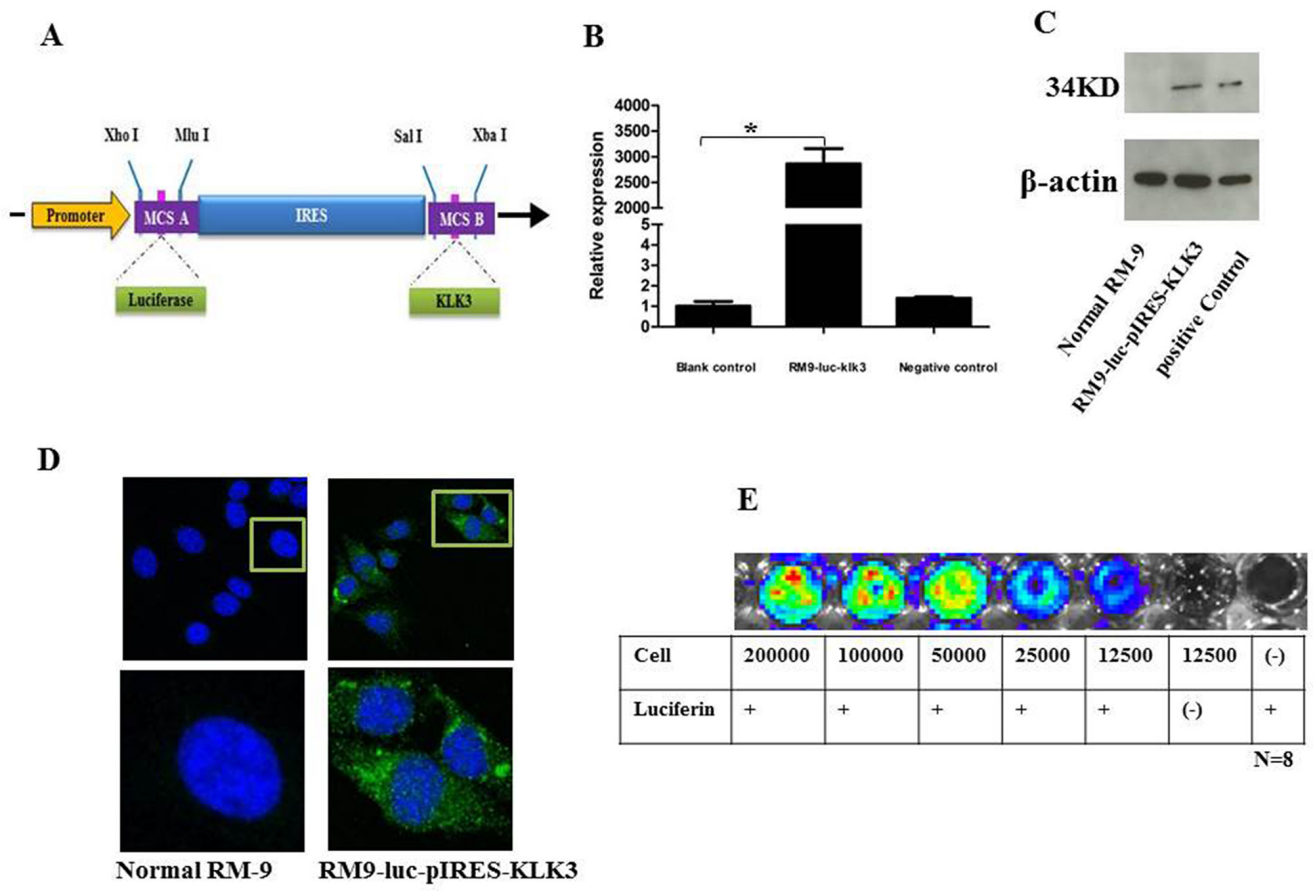
Construction and verification of the new recombinant cell line RM9-luc-pIRES-KLK3 **A.** Schematic representation of the construction of plasmid Luc-pIRES-KLK3. *Luc* was obtained by enzyme digestion of plasmid pGL3-basic. *KLK3* were obtained through RT-PCR. The IRES element was chosen to connect upstream *Luc* and downstream *KLK3*. Restriction enzyme cutting sites included Xho I, Mlu I, Sal I, and Xba I. RT-PCR, reverse transcription polymerase chain reaction; IRES, internal ribosome entry site; pCMV, cytomegalovirus promoter **B.** KLK3 mRNA expression level in RM9-Luc-pIRES-KLK3 cell line * vs. control, *p* < 0.001 **C.** Western blotting analysis for prostate specific antigen expression Normal RM9 cells served as negative control (left) and LNCaP cells served as positive control (right) for RM9-Luc-pIRES-KLK3 cells (middle). Beta-actin levels served as loading control. **D.** Immunofluorescence (IF) staining using PSA on RM9-Luc-pIRES-KLK3 cell line. PSA (green), cell nuclear (pseudo-colored blue). RM9 cells are stained as a negative control which shown on the left. Images were captured using a 40× oil objective. **E.** *In vitro* bioluminescence imaging of RM9-Luc-pIRES-KLK3 cells In a 96-well plate, cells were serially diluted from 200,000 to 12,500 cells. Luciferin 100 μl (150 μg/ml) was added to all wells, except for blank controls, and the plate was subjected to imaging for 1 min. Bioluminescence per well was quantified in photons per second. Expression was positively correlated with cell counts.

Using quantitative polymerase chain reaction (qRT–PCR), we confirmed that KLK3 mRNA level was significantly higher in the RM9-Luc-pIRES-KLK3 cell line (2858.09 ± 300.27) than in the blank control group (1 ± 0.24, p = 0.000) and in the negative group (1.39 ± 0.07, p = 0.000). This indicated a remarkable transfection efficiency of *KLK3* (Figure 1B).

The measurement of PSA expression in RM9-Luc-pIRES-KLK3 cells by Western blot analysis is shown in Figure 1C and was confirmed by immunofluorescence (IF) (Figure 1D). The Cell Proliferation assay showed that potential proliferation was the same between the RM9-Luc-pIRES-KLK3 and normal RM9 subsets (data not shown). *In vivo* imaging system (IVIS)-200 detection of luciferase expression showed a positive correlation between the intensity of bioluminescence and cell count (Figure 1E).

### Monitoring of tumorigenicity by RM9-Luc-pIRES-KLK3 cells

Subsequently, we developed subcutaneous, prostate orthotopic xenograft, and lung metastasis models based on mouse prostate cancer RM9-Luc-pIRES-KLK3 cells.

**Figure 2:**
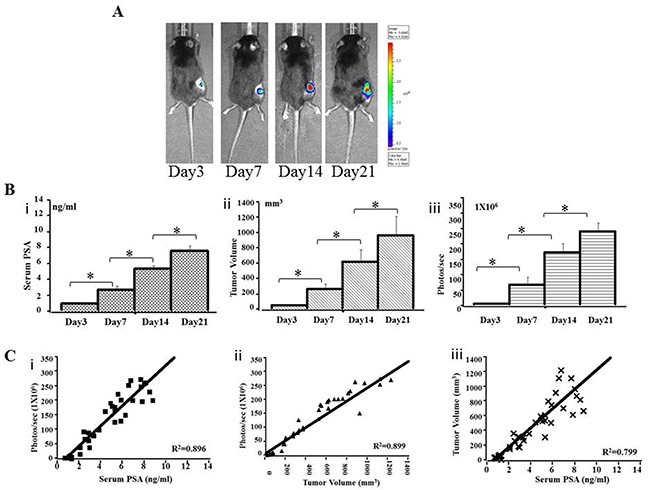
Preclinical subcutaneous mouse models using RM9-Luc-pIRES-KLK3 cells (n = 7) **A.** Bioluminescence images in a representative subcutaneous mouse model. Animals were imaged weekly for 3 weeks from the third day after injection. **B.** Tumor progression was monitored by serum PSA (ng/ml) (i), tumor volume (mm^3^) (ii) and bioluminescent imaging (photos/s) (iii) on days 3, 7, 14, and 21 after RM9-Luc-pIRES-KLK3 injection. Mean values were analyzed among groups. * p < 0.05 **C.** Comparison between bioluminescence intensity and the current methods of monitoring tumor size and serum PSA levels. The observed bioluminescent imaging (photos/s) was proportional to the serum PSA (ng/ml) (R^2^ coefficient: 0.896) (i) and tumor volume (mm^3^) (R^2^ coefficient: 0.899) (ii), R^2^ coefficient: 0.799 was proportional between serum PSA (ng/ml) and tumor volume (mm^3^) (iii).

**Figure 3:**
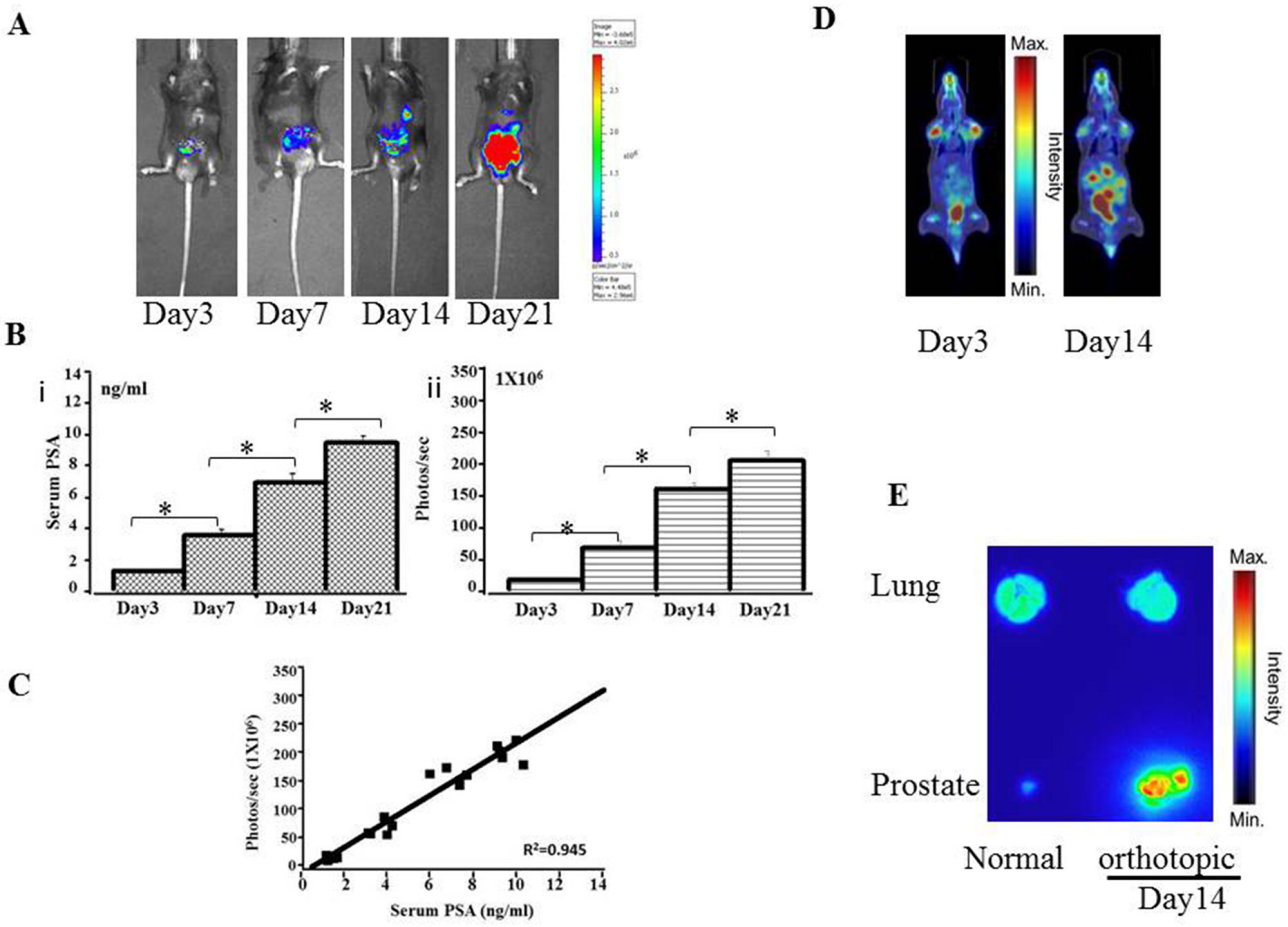
Preclinical orthotopic mouse models using RM9-Luc-pIRES-KLK3 cells (n = 7) **A.** Bioluminescence images in a representative orthotopic mouse model. **B.** Tumor progression was monitored by evaluating serum PSA levels (ng/mL) (i) and bioluminescence intensity (photos/s) (ii) on days 3, 7, 14, and 21 after RM9-Luc-pIRES-KLK3 was injected into the prostate. Error bars represent standard error of mean. Mean values were analyzed among groups. *p < 0.05 **C.** Comparison between bioluminescence intensity and current methods of monitoring tumor size and serum PSA levels. The observed bioluminescent imaging (photos/sec) was proportional to the serum PSA (ng/ml) (R^2^ coefficient: 0.945). **D.** ^18F^FDG-PET images of orthotopic tumor-bearing mice on day 3 and day 14 after RM9-Luc-pIRES-KLK3 was injected into the prostate. **E.** Anatomical autoradiography images of the prostate and lung in the orthotopic prostate cancer model on days 3 and 14 after RM9-Luc-pIRES-KLK3 injection.

**Figure 4:**
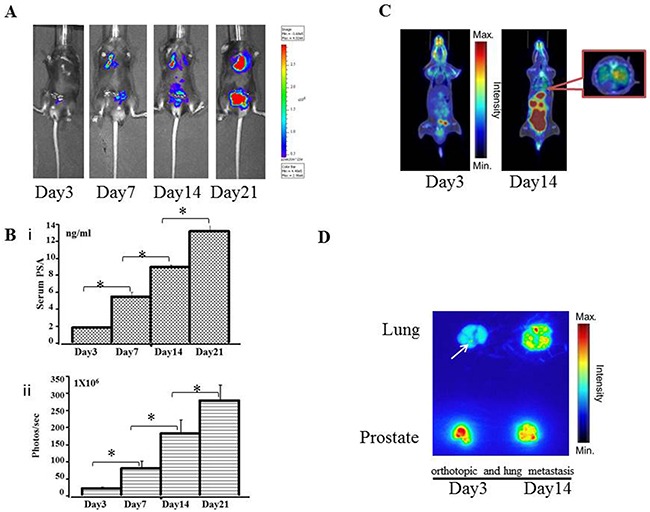
Preclinical orthotopic and lung metastasis mouse models using RM9-Luc-pIRES-KLK3 cells (n = 7) **A.** Bioluminescence images in a representative orthotopic and lung metastasis mouse model. **B.** Tumor progression was monitored by evaluating serum PSA level (ng/mL) (i) and bioluminescence intensity (photos/s) (ii) on days 3, 7, 14, and 21 after RM9-Luc-pIRES-KLK3 was injected into the prostate. Error bars represent standard error of mean. Mean values were analyzed among groups. *p < 0.05 **C.** ^18^F-FDG PET images of orthotopic tumor-bearing mice at day 3 and day 14 after RM9-Luc-pIRES-KLK3 was injected into the prostate and tail vein **D.** Anatomical autoradiography images of the prostate and lung in the orthotopic prostate cancer model at days 3 and 14 after RM9-Luc-pIRES-KLK3 injection. In the metastasis model, micrometastatic points were sensitively detected on day 3 after RM9-Luc-pIRES-KLK3 injection.

A pre-experiment, real-time IVIS imaging analysis of tumor growth revealed that RM9-Luc-pIRES-KLK3 cells readily grew in mice 3 days after injection. Figure 2A shows representative images of mice subcutaneous model in which the intensity of bioluminescence visibly increased over time. Measurement and analysis of serum PSA (Figures 2B i), tumor volume (Figures 2B ii), and bioluminescent imaging (Figures 2B iii) showed that bioluminescent imaging was proportional to serum PSA level (R^2^ coefficient = 0.896) (Figures 2C i) and tumor volume (R^2^ coefficient = 0.899) (Figures 2C ii), R^2^ coefficient: 0.799 was proportional between serum PSA (ng/ml) and tumor volume (mm^3^) (Figures 2C iii). After implantation and inoculation of RM9-Luc-pIRES-KLK3 cells in the prostate only (Figure 3A) for mice orthotopic prostate cancer model and after the inoculation of cells into prostate and tail vein for mice orthotopic prostate cancer with metastatic model, micrometastatic deposits were detected by real-time bioluminescent imaging 7 days after transplantation; lung metastases spread exponentially on dynamic observation in the metastatic model (Figure 4A). Moreover, weekly monitoring showed increased serum PSA levels (i) and bioluminescent imaging (ii) over time in the orthotopic prostate cancer model (Figure 3B) and lung metastasis model (Figure 4B). In the orthotopic prostate cancer model, there was a positive correlation between bioluminescent imaging and the serum PSA level (R^2^ coefficient = 0.945) (Figure 3C).

### Biodistribution analysis by PET-CT and autoradiography

After transplantation of RM9-Luc-pIRES-KLK3 cells in mice for 3 and 14 days, positron emission tomography–computed tomography (PET–CT) scan showed increased real-time uptake of the radiotracer (Figure 3D and 4C). The abnormal areas detected on PET molecular imaging corresponded with those detected by bioluminescence imaging (BLI) and were finally confirmed by histopathology.

Anatomical autoradiography images of the prostate and lungs in the orthotopic prostate cancer and lung metastasis models on days 3 and 14 after PET-CT scan are shown in Figure 3E and Figure 4D, respectively. At each time point, high tracer uptake was detected in the tumor. In particular, the area of micrometastasis was sensitively detected on day 3 in the lung metastasis model (Figure 4D).

### Therapeutic efficacy of immune checkpoint CTLA-4 blockade in a prostate cancer model using RM9-Luc-pIRES-KLK3 cells

**Figure 5:**
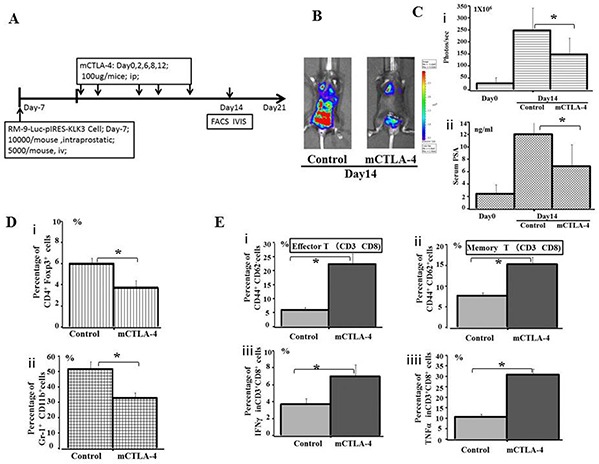
Evaluation of the effect of immunotherapy by immune checkpoint CTLA-4 blockade **A.** The plan for therapeutic effect on mice CTLA-4 blockade. **B.** Bioluminescence images of mice from each treatment group are shown 14 days after treatment. RM9-Luc-pIRES-KLK3 cells in tumors were imaged after luciferin injection using the IVIS instrument. Left panel shows the control group, whereas the right panel displays the mice CTLA-4 blockade group. **C.** Tumor volume (i) was determined by region-of-interest analysis of total photons per second. Serum PSA levels (ng/mL) (ii) were determined by ELISA from the groups of CTLA-4 blockade and control. Mean values were analyzed among groups. * CTLA-4 blockade vs. control (p < 0.05). **D.** The percentage of peripheral CD4^+^Foxp3^+^ Treg cells (i) and Gr-1^+^CD11b^+^ MDSC cells (ii) among the total number of lymphocytes, as quantified by FACS analysis, are shown for the indicated groups and times. Mean values were analyzed among groups. * CTLA-4 blockade vs. control group (p < 0.05). **E.** The percentage of peripheral CD44^+^CD62^−^ Effector T cells (i), CD44^+^CD62^+^ Memory T cells (ii), IFN-γ (iii), and TNFα (iv) in population of CD3^+^CD8^+^ cells were quantified by FACS analysis and are shown for the indicated groups and times. Mean values were analyzed among groups. *CTLA-4 blockade vs. control group (p < 0.05).

The therapeutic potential of immune checkpoint CTLA-4 blockade was validated *in vivo* using orthotopic and lung metastatic models. RM9-Luc-pIRES-KLK3 cells were transplanted into the prostate and tail vein of C57BL/6 mice; thereafter, CTLA-4 blockade was administered intraperitoneally at a dose of 100 μg per mice on days 0, 2, 6, 8 and 12 (Figure 5A). Figure 5B shows the BLI of the groups of mice with CTLA-4 blockade compared with the negative control after 2 weeks treatment.

As shown in Figure 5C, tumor volume (Figure 5C i), as analyzed by the IVIS instrument, was significantly smaller after mice CTLA-4 blockade, compared with the control groups (*p* = 0.001). Serum PSA (Figure 5C ii) significantly inhibited tumor infiltration 14 days after treatment (*p* = 0.017).

After 2 weeks of treatment, the proportion of CD4^+^ Foxp3^+^ Treg cells (Figure 5D i) and Gr-1^+^CD11b^+^MDSC cells (Figure 5D ii) were significantly downregulated in the CTLA-4 blockade group of mice compared with the phosphate-buffered saline (PBS)-treated group. Meanwhile, the proportion of CD44^+^CD62^−^ effector T cells (Figure 5E i) and memory T cells (Figure 5E ii) on CD3^+^CD8^+^ cells were significantly increased by >2–3 times after CTLA-4 blockade compared with the control treatment. Production of IFN-γ (Figure 5E iii) and TNFα (Figure 5E iv) by CD3^+^CD8^+^ cells was elevated in the CTLA-4 blockade group of mice compared with the control, suggesting prolonged survival in former treatment group. In addition, the analysis of liver and other body tissue specimens indicated no histopathological damage in any of the treatment groups (data not shown).

## DISCUSSION

PSA is recognized as a sensitive biomarker and is one of the most important indices for clinical diagnosis and prognosis of prostate cancer. When the blood–epithelium barrier is destroyed, PSA secreted by cancer cells increases and enters the blood directly to cause metastatic prostate cancer [11, 12].

Within an organism, luciferase catalyzes luciferin to cause a luminous oxidation reaction that may be detected as signals on the noninvasive IVIS living imaging system. At present, this system is favored by many scholars for its high efficiency, sensitivity, rapidity, and minimal invasiveness [12–15]. In this study, the internal ribosome entry site (IRES) element, which is the functional site of the internal ribosome [16], was chosen to connect the upstream luciferase genes and the downstream KLK3 genes by transcribing luciferase and KLK3 into a single-chain mRNA. Although transcription is simultaneous, KLK3 and luciferase can be independently translated and expressed. Therefore, genetic engineering was combined with bioluminescence to construct a new cell line that was detectable by intravital imaging using a strongly expressed tumor biomarker. Using these cells, different types of mouse models were established to simultaneously monitor tumor volume, intensity of fluorescence, and PSA level.

BLI and PET are the two main methods that can determine the growth and progression of carcinomatous tumors in mouse models by real-time imaging [14, 15]. Micrometastatic deposits in the lung were detected using real-time bioluminescence imaging 7 days after cell-transplantation in mice orthotopic prostate cancer with metastatic model. PET scan is a versatile and non-invasive tool that not only provides a three-dimensional (3D) image of tissue function but also obtains better imaging of lymph node and distant metastases; therefore, PET could identify tumor tissue necrosis even before the lesion extruded the organ surface [14, 15]. The usual tracer of PET is ^18^F-fluorodeoxyglucose (FDG), an analogue of glucose whose uptake is regulated by glucose transporters and hexokinase activity; this is the most common and standard tracer used to detect cancer metastasis by PET scan. Therefore, ^18^F-FDG PET has been recently indicated as a possible means to assess the biological activity of advanced cancers and to evaluate a specific subgroup of patients carrying lesions with aggressive phenotypes [14, 17]. However, ^18^F-FDG PET has poor sensitivity for soft tissue tumors. In contrast, bioluminescence is limited by its poor spatial resolution.

Considering these advantages and limitations of PET and BLI, we combined these two methods in an animal model to detect beginning tumor lesions by optical imaging and then increasing their resolution by PET scan. In the present study, we found that although BLI could detect lesions earlier than PET scan, its spatial conformation of lesions was not as much as PET, especially, in the identification of lung metastases. PET scan could provide a series view of sagittal, coronal, and transverse sections on the lung lesion, which could be reconstructed as a three-dimensional image to help understand the size and extent of the metastasis. On the other hand, it excluded necrotic regions of the tumor, scar tissue, calcification, and other metabolically inactive tissues via measure and contrast the background metabolism in normal muscle and in normal parenchyma; therefore, both BLI and PET are needed to obtain sufficient information on tumors. Since the emergence of immunotherapies, such as therapeutic cancer vaccines and immune checkpoint inhibitors, cancer treatment has stepped into a new era [5, 6]. The first in class approved immune checkpoint inhibitor is ipilimumab, an anti-CTLA-4 (cytotoxic T lymphocyte antigen-4) mAb. In 2011, the US Food and Drug Administration (FDA) approved ipilimumab for metastatic melanoma. Till now, it was the most mature checkpoint blockade. Recently, immune-based combination therapy of prostate cancer is being developed in full swing by preclinical and clinical studies [18, 19]. It is extremely important to examine the safety, efficacy, and side effects of combination therapy by analyzing the immune status.

CD11b^+^ Gr-1^+^ MDSC cells are a heterogeneous population comprising immature myeloid cells and myeloid progenitor cells that suppress immune responses by a variety of mechanisms. CD4^+^ Foxp3^+^ Treg cells play an important role in immune evasion and inhibit the efficacy of vaccine therapy [18–21]. Therefore, it is obvious that successful cancer immunotherapy will require the inhibition of the immunosuppressive effects of these cell populations. Furthermore, the proportion of CD44+CD62− effector and memory T cells on CD3+CD8+ cells were also evaluated in both groups, as well as the production of IFN-γ and TNFα by CD3+CD8+ cells. In contrast, the CTLA-4 blockade treatment group continued showing a high proportion of effector and memory T cells, and in terms of cytokines, early detection showed the same trend, but after one period of increase, we found that cytokines gradually reduced (data not shown); the specific reasons and mechanisms are not yet clear. Whether this is one of the reasons for immunotherapy failure still needs further research. However, our results on CTLA-4 blockade provided potential reasons for the failure of immunotherapy for cancer and suggested the need for future studies on immunomodulators against cancer [6, 22].

However, there are still some drawbacks in the present study. First, we did not describe the model with bone metastasis, which is common in human prostate cancer. Second, we assessed limited parameters to assess the immune status in mice. In future, we will try to construct the bone metastasis model with this new cell line and expand the assessment methodology on the immune status.

In summary, the use of combined bioluminescence and tumor biomarker system on animal model cancer cell lines substantially advances the preclinical researches on immunotherapy and imaging for antitumor drug development. Our study confirmed by real-time imaging that changes in immune function and tumor biomarkers can be analyzed by immune checkpoint blockade in prostate cancer orthotopic and lung metastasis models in immunocompetent mice. The results may pave the way for establishing the basic knowledge on immune checkpoint blockade and new drugs to clinical trial application.

## MATERIALS AND METHODS

### Tumor cell lines

The human prostate cancer cell line LNCaP was purchased from American Type Culture Collection (Rockvile, MD). The C57BL/6 mouse prostate cancer cell line RM-9 was kindly provided by Dr. T.C. Thompson of The University of Texas, Houston, TX. The cell lines were cultured in RPMI-1640 with 10% fetal bovine serum (Gibco, Invitrogen, Carlsbad, USA) and maintained at 37°C in a humidified atmosphere containing 5% CO_2_.

### Plasmid construction and transfection and cell proliferation assay

Eukaryotic expression vector pIRES and pGL3-basic vector were purchased from Clontech Laboratories, Inc. (Mountain View, USA). PCR primers were designed according to the GenBank expression of human PSA protein (*KLK3*) sequences (GI:22208990). Luciferase gene sequences (GI:13195703) were obtained from pGL3-basic vector by endonuclease digestion. Using reverse transcriptase PCR (RT-PCR) and PCR amplification technologies, a Luc-pIRES-KLK3 plasmid was constructed (Figure 1A) for transfectioninto RM9 cells for stable expression in monoclonal strains; G418 was successfully used to filter out the RM9-Luc-pIRES-KLK3 monoclonal cell line. The Cell Proliferation assay showed that potential proliferation was the same between the RM9-Luc-pIRES-KLK3 and normal RM9 subsets by The Cell Proliferation Kit II (XTT, Roche, Basel, Switzerland).

### Expression of prostate-specific antigen

The cell line was characterized *in vitro* to test for the expression of human PSA by Western blotting and IF. For Western blotting, the total protein from the cloned cells was extracted and its concentration was determined using a bicinchoninic acid assay (Thermo Scientific). Protein samples were run on 12% SDS-PAGE gels and were transferred to polyvinyldene difluoride membranes (Bio-Rad Laboratories, Inc.). The membranes were incubated overnight using rabbit anti-human PSA antibody (Cell Signaling Technology, Danvers, USA) as a primary antibody at 1000× dilution. Next, the membranes were washed and immediately incubated with anti-rabbit horseradish peroxidase-conjugated secondary antibodies.

For IF, pre-blocking clone cells were incubated with the primary antibodies overnight in a humidified chamber at 4°C. All specimens were then washed with PBS and immediately incubated with fluorochrome-conjugated secondary antibody [Alexa Fluor 488 goat anti-rabbit IgG (green), Life Technologies] diluted in antibody dilution buffer for 1 h at room temperature in a dark environment. Slides were covered with mounting medium with DAPI (blue, Vector Laboratories, Inc., Burlingame, CA, USA). Negative controls were performed on RM9 cell lines. Confocal laser scanning microscopy was performed with Zeiss LSM 780 (Carl Zeiss, Oberkochen, Germany) using 40× oil objective lens, as indicated.

### Quantitative real-time PCR

Total RNA was isolated by TRIzol reagent (Invitrogen, Grand Island, NY, USA) and cDNA was obtained by RT-PCR. Approximately 1 μl (1μg) of total RNA was added to 12 μL of moloney murine leukemia virus reverse transcriptase (Promega, Madison, WI, USA). The reaction mixture was prepared following the manufacturer's instructions. All primers were synthesized by Invitrogen and were checked for specificity before use. Quantitative RT-PCR was performed under the following reaction conditions: stage 1, 50°C for 2 min (Rep 1); stage 2, 95°C for 2 min; stage 3, 95°C for 15 s and then 60°C for 32 s (Reps 40). Thereafter, comparative CT method (2^−ΔΔCT^) was adopted to evaluate the relative quantitation. 18srRNA was used as internal reference.

### In vitro bioluminescence detection

For in vitro BLI, RM9-Luc-pIRES-KLK3 cell concentrations, ranging from 2 × 10^5^ to 1.25 × 10^4^ cells, were serially diluted in 96-well plates (Costar, Corning, USA); images were obtained after the addition of D-luciferin (Biosynth, Naperville, USA) to the media. Wells containing medium only and RM9 cells served as blank control and negative control, respectively. D-luciferin 100 μl (150 μg/ml) was added to all wells. Prior to imaging, cells were incubated at 37°C in a humidified atmosphere containing 5% CO_2_ for 10 min. Images were captured using IVIS-200 (Xenogen, Alameda, USA) and analyzed with Living Image software (Xenogen Corporation). Luciferase activity was monitored by the measurement of bioluminescence intensity. Results were reported as the average of three independent experiments.

### Mouse model

Male C57BL/6 mice (6–8 weeks old) were purchased from SLC Inc. (Hamamatsu, Japan) and were maintained in a specific pathogen-free environment at the laboratory animal center of Okayama University. They were allowed to adapt to their environment for 2 weeks before the experiments. The animals were housed and handled in accordance with the Okayama University Animal Research Committee Guidelines. For all surgical procedures, mice were anesthetized with an intraperitoneal injection of 10% chloral hydrate at a dose of 300 mg/kg. Anesthetized animals received subcutaneous injection of RM9-Luc-pIRES-KLK3 cells as follows: 2.0 × 10^5^ cells in a 100-μl re-suspension below the dorsal flank; 1.0 × 10^4^ cells in 10-μl re-suspension into the dorsolateral prostate for the orthotopic model; and 0.5 × 10^4^ cells in a 100-μl re-suspension into the tail vein for the lung metastasis model. Mice were observed twice weekly after inoculation. Tumor volumes in the subcutaneous tissue were calculated from two perpendicular tumor diameters (d), according to the following formula for the volume of an ellipse: V = π/6(d1 × d2)^3/2^.

### Bioluminescence imaging

The animals underwent imaging at a peak time of 2 min after intraperitoneal injection of D-luciferin (150 mg/kg) via IVIS-200 (Xenogen, Alameda, USA); appropriate exposure times and sensitivity settings were used to avoid saturation. Image processing was performed using the Living Image software (Xenogen) by counting the total number of photons per second for each region of interest in a tumor, with appropriate background subtraction.

### Serum PSA analysis using ELISA

For the PSA assay, 50 μl of serum was analyzed using a human PSA enzyme-linked immune sorbent assay kit (ELISA, ANOGEN-YES BIOTECH, Ontario, Canada). The optical density was read at 450 nm using a microtiter plate; data were collected and assayed using Microplate Manager 5.0 PC software (BIO-RAD, California, USA).

### ^18^F-FDG PET and autoradiography imaging

A small-animal PET–CT system (Clairvivo PET, SHIMADZU, Kyoto, Japan) was used with the tracer ^18^F-FDGthat was injected at a dose of 4.2 ± 0.4 MBq in the tail vein of mice. Animals were anesthetized 10 mins before radioligand administration. PET scanning started at 60 min after tracer injection and lasted for 30 min. After the last time of *in vivo* PET scanning, tumor tissues were removed *en bloc* and underwent *ex vivo* PET scanning. All PET imaging data were acquired in list mode; PET scan images were reconstructed using filtered back projection and an iterative 3D reconstruction algorithm (maximum a posteriori). CT images were reconstructed using a Feldkamp convolution–back projection algorithm. ^18^F-FDG PET and CT data were then analyzed and quantified by AMIDE software, version 0.7.154 [12]. The mice underwent euthanasia after PET measurements. The entire prostate and lung were immediately removed and contacted with an imaging plate, which was scanned using a biomolecular imager (Typhoon™ FLA 7000; GE Healthcare Life Science, Co., Ltd.).

### Immune checkpoint blockade treatment and flow cytometry analysis in vivo

Mice from the metastasis models were randomly divided into two groups and were injected intraperitoneally with 100 μg of anti-CTLA-4 (clone 9H10, BioXCell) or isometric PBS on days 0, 2, 6, 8 and 12, as previously reported [13–14]. Peripheral blood (200 μl) from each mouse was collected in a centrifuge tube with ethylenediaminetetraacetic acid and were treated with FACS lysing solution before incubating for 1 h at 4°C with fluorescein isothiocyanate-labeled anti-mouse CD11b, Foxp3, and CD8 antibody; phycoerythrin (PE)-labeled anti-mouse Gr-1, CD4, and CD3 antibody; allophycocyanin-labeled anti-mouse-CD44; PE-Cyanine7-labeled anti-mouse CD62; PerCP-Cyanine5.5-labeled anti-mouse IFN-γ; and PE-eFluor 610-labeled anti-mouse-TNFα (eBioscience, San Diego, USA). Labeled samples were washed twice with cold PBS and then re-suspended in 200 μl of cold PBS before analyzing on fluorescence-activated cell sorter flow cytometer (Calibur; BD Biosciences, San Jose, USA) by gating on lymphocytes.

### Statistical analyses

The data were reported as mean ± standard deviation. Correlation analysis was used to describe the relationship among bioluminescence intensity, tumor volume, and serum PSA levels. One-way analysis of variance followed by Bonferroni's *post hoc* comparison tests was performed to compare the different models. Paired Student's t-test was performed for dependent variables. Statistical significance was set at p < 0.05.
